# The Medical Education Partnership Initiative (MEPI), a collaborative paradigm for institutional and human resources capacity building between high- and low- and middle-income countries: the Mozambique experience

**DOI:** 10.1080/16549716.2017.1272879

**Published:** 2017-01-27

**Authors:** Emilia Virginia Noormahomed, Carla Carrilho, Mamudo Ismail, Sérgio Noormahomed, Alcido Nguenha, Constance A. Benson, Ana Olga Mocumbi, Robert T. Schooley

**Affiliations:** ^a^ Department of Microbiology, Faculty of Medicine, Universidade Eduardo Mondlane, Maputo, Mozambique; ^b^ Department of Medicine, Infectious Disease Division, University of California, San Diego, CA, USA; ^c^ Reitoria, Universidade Lurio, Nampula, Mozambique; ^d^ Department of Planning and Cooperation, Mozambique Institute for Health Education and Research (MIHER/MEPI), Maputo, Mozambique; ^e^ Department of Pathology, Faculty of Medicine, Universidade Eduardo Mondlane, Maputo, Mozambique; ^f^ Department of Administration and Finance, Mozambique Institute for Health and Research (MIHER/MEPI), Maputo, Mozambique; ^g^ Department of Non Communicable Diseases, Instituto Nacional de Saúde, Maputo, Mozambique; ^h^ Department of Medicine, Faculty of Medicine, Universidade Eduardo Mondlane, Maputo, Mozambique

**Keywords:** North–South collaboration, South–South collaboration, Medical Education Partnership Initiative, capacity building

## Abstract

**Background:** Collaborations among researchers based in lower and middle income countries (LMICs) and high income countries (HICs) have made major discoveries related to diseases disproportionately affecting LMICs and have been vital to the development of research communities in LMICs. Such collaborations have generally been scientifically and structurally driven by HICs.

**Objectives:** In this report we outline a paradigm shift in collaboration, exemplified by the Medical Education Partnership Initiative (MEPI), in which the formulation of priorities and administrative infrastructure reside in the LMIC.

**Methods:** This descriptive report outlines the critical features of the MEPI partnership.

**Results:** In the MEPI, LMIC program partners translate broad program goals and define metrics into priorities that are tailored to local conditions. Program funds flow to a LMIC-based leadership group that contracts with peers from HICs to provide technical and scientific advice and consultation in a 'reverse funds flow' model. Emphasis is also placed on strengthening administrative capacity within LMIC institutions. A rigorous monitoring and evaluation process modifies program priorities on the basis of evolving opportunities to maximize program impact.

**Conclusions:** Vesting LMIC partners with the responsibility for program leadership, and building administrative and fiscal capacity in LMIC institutions substantially enhances program relevance, impact and sustainability.

## Background

### Traditional collaborations between Northern and Southern research partners

Traditional collaborations between Northern and Southern research partners. Health research collaborations between institutions from high-income countries (HICs) and low- and middle-income countries (LMICs), designated in this paper as countries from the North and South, respectively, have taken several forms and have evolved over time. Structural differences in these relationships have been driven by wide North–South disparities that vary according to the participating country and institutions, the socioeconomic environments surrounding collaborating partners, the projects and researchers involved, and to some extent the specific research issue under study [1–7].

During the 1960s research cooperation consisted mainly of technical assistance from the North to the South and was directed mainly at training young academics from Southern countries in institutions of Northern countries [5]. While this facilitated training individual scientists from Southern institutions, those who were trained in the North faced several challenges when they completed their training and returned to their home countries. Such challenges included working conditions that lacked the necessary infrastructure, equipment, and financial underpinnings to apply the knowledge and skills gained in their training experiences after returning to LMICs. Returning trainees often found that research areas in which they were trained were of limited relevance to their home countries and they were also demotivated by low salaries. The subsequent brain drain of many talented Africans to Northern countries aggravated the shortage of human research resources in Africa [5,8].

In the 1970s there were attempts to strengthen research capacity in developing countries, and especially in improving the access to scientific information from the North. Nonetheless, research agendas were in most cases largely developed by Northern collaborators and left to Southern collaborators to execute. The work was often undertaken as part of advanced degree training [5,9–12]. Collaborations between North and South were usually seen as an association in which the Southern partner was viewed as the ‘receiver’ and the Northern partner as the ‘giver’. This left the perception of Southern weakness rather than Southern skill deficits. The benefits derived by the North from the relationship often remained hidden [4,9].

In more recent years there has been a gradual evolution in the relationships among Northern and Southern partners in the direction of more bona fide partnerships based on mutual interest, responsibility, trust, and transparency. Collaborative research networks have developed with substantially more parity between Northern and Southern participants. This evolution has occurred more demonstrably within some funding agencies than others. Despite this progress, in many cases these collaborations are still not conducted with each partner on an equal footing. In his Berlin 2014 Africa Day speech entitled ‘On the Impossibility of Speaking of Africa’, the former President of the Federal Republic of Germany, Professor Horst Kohler, stated that ‘many colonial and post colonial attitudes persist to this day, sometimes latent and unsuspected but sometimes quite overtly.’ For a number of reasons substantial changes are still required within many funding agencies to restructure longstanding relationships that have largely defined the culture of research collaborations between researchers from Northern and Southern countries. Among these agencies, the agenda and priorities of research, the management, and the fiscal administration are still defined largely by the North [4,5,7,9].

There are a few existing outstanding examples of mutually beneficial North–South research collaborations, for example the Ghanaian–Dutch partnership, the European and Developing Countries Clinical Trials Partnership (EDCTP), the Rwanda Human Resources for Health Program, The Global Health Service Partnership, and the United States (US) President’s Emergency Plan For AIDS Relief (PEPFAR)-supported Medical Education Partnership Initiative (MEPI) that we will examine in this manuscript [7–9,11,12].

The objective of this paper is to: describe some of the outcomes of the MEPI program in Mozambique and other African countries; critically analyze and compare the main features of this collaboration with those of traditional models of collaboration between North and South institutions; and describe strategies developed to sustain the aims of the program beyond the initial 5 years of financial support.

## MEPI overview: implementation, aims, and achievements

With support from the US Department of State and the US National Institutes of Health (NIH) and leadership from a Council of Principal Investigators drawn from 12 sub-Saharan African countries, the MEPI was launched in 2010 to develop transformative models in medical education and to build research and bioinformatics capacity to dramatically and sustainably increase the training and retention of physicians and scientists where they are most needed. One hundred and thirty million US dollars (USD) were invested over 5 years in 13 dynamic partnerships between African and US institutions [13]. The Universidade Eduardo Mondlane (UEM) in Mozambique, through a partnership formed in 2008 with University of California, San Diego (UCSD), was one of the African universities that was awarded $10,620,000.00 for 5 years under the MEPI program. UEM in turn formed a consortium with the Faculties of Health Sciences in two new Mozambican universities: the Universidade Lurio (UniLurio) and the Universidade Zambeze (UniZambeze), located in Nampula and Sofala provinces, respectively, both of which are underserved regions in central and northern Mozambique. This consortium jointly designed and implemented the MEPI program, in collaboration with UCSD as a technical partner.

The MEPI program is built around five core themes. These include: (1) strengthening training and research to enhance the capacity and quality of physicians trained; (2) developing a critical mass of African researchers to address the most pressing health problems in their own countries; (3) retaining health workers where they are most needed; (4) developing communities of practice to strengthen partnerships to address common areas of interest; and (5) achieving sustainable institutional development to ensure that MEPI accomplishments will continue beyond the initial budget period.

Participating schools outlined approaches that they believed were best suited to address these themes in competitively reviewed grant applications. Improvement of medical education was addressed by the adoption of more innovative curricula and by substantial efforts at faculty development with a goal of increasing the number of graduates who could enter faculty positions and improving the quality of trained physicians. Three Master’s degree programs were created at UniLurio to promote faculty development by enhancing teaching and research capabilities, promoting professional satisfaction, and creating vibrant communities of practice with common goals, and to retain faculty and other health professionals at UniLurio to populate faculty positions in the new medical school. To date 24 students have completed their dissertations: 17 in the Master of Health Professional Education and 7 in the Master of Tropical Medicine and International Health.

In order to retain physicians where they are most needed, some programs across the participating MEPI countries developed community-based sites and recruited students from rural areas [14–16]. Whenever possible multiple medical schools in each participating country were engaged to create synergies [11,17,18]. Large investments were made to enhance the informatics infrastructure as an essential tool for distance learning, to decentralize training to rural sites, and to allow access to digital medical textbooks and other scientific literature. Innovative digital platforms enabled electronic transmission of clinical and laboratory data and the downloading of treatment guidelines to smart phones and other mobile electronic devices.

Geographical barriers to travel were overcome by creating collaborative teaching teams among health care professionals connected by electronic distance learning (eLearning) programs [11,14,16,19–21]. As an example, information technology infrastructure was established through SEACOM (a fiber optic cable along Africa’s east coast) to provide Internet connections within the Faculty of Medicine (FoM) at UEM, the Medicine, Surgery, and Pediatric wards, the Emergency Department, and the teaching conference rooms and clinical laboratory at Maputo Central Hospital (MCH). Electronic virtual libraries were established in the Departments of Medicine and Surgery at MCH and at FoM-UEM, with installation of 60 computers and 48 tablets across these sites allowing free access to medical literature for students and health care workers and to facilitate point-of-care access to the medical literature and enhance health information technology skills.

Research capacity was strengthened by the development of structured mentorship programs. Several didactic courses such as research methodology and grant and manuscript writing were developed and provided to the research community on a regular basis [11,21]. Since the beginning of the MEPI program in Mozambique, 28 research projects have been generated and funded by different entities, including the US NIH, and 37 papers have been published. The complete list of our publications can be seen at http://www.ncbi.nlm.nih.gov/sites/myncbi/emilia.noormahomed.1/bibliography/44995742/public/?sort=date&direction=ascending.

The development of research capacity provided additional fiscal resources for faculty and students as well as the institutional cohesiveness that is essential to combat both internal and external brain drain. Emphasis was also placed on the development of institutional capacity for sustainable development so that the legacy of this program could last for decades.

## Contrasts between traditional models of collaboration and MEPI

**Figure 1.**
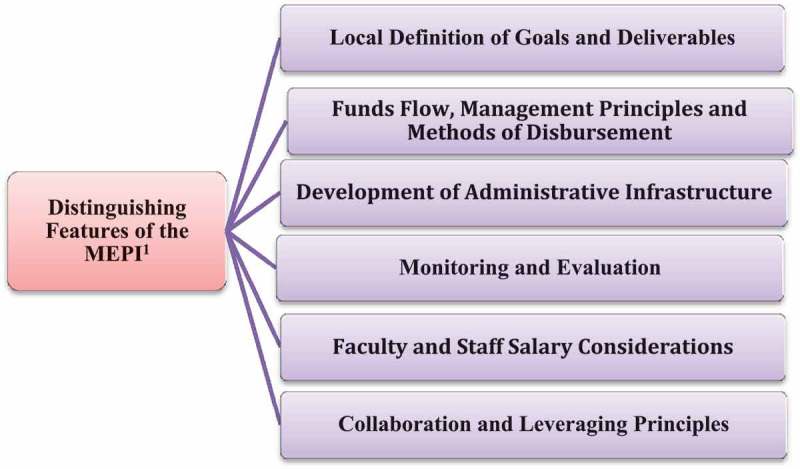
MEPI partnership main features analyzed. Note: ^1^MEPI: Medical Education Partnership Initiative.

Several key features differentiate the MEPI from the traditional model of collaboration. See Tables 1 and 2. These include: (1) locally defined goals and deliverables; (2) the approach taken to funds flow, disbursement, and management principles; (3) detailed monitoring and evaluation (M&E); (4) the emphasis on development of a robust administrative infrastructure; (5) identified effort-based supplemental funding for faculty and staff; and (6) broad collaboration and aggressive leveraging (see Figure 1). In this paper we will delineate several key features of the MEPI that we believe show this program to be a paradigm shift in the collaboration between Northern and Southern institutions.

**Table 1.** Contrasts between traditional model of collaboration and MEPI^1^.

Definition of goals and deliverables	Traditional model Funders usually set priorities and specify deliverables.	MEPI model Funders lay out programmatic goals.
	Funders usually develop and design the project.	Local investigators delineate goals and specific aims tailored to local needs and opportunities.
	Local personnel are essentially ‘hired’ by the funded partner from the country of the granting agency.	The research team jointly develops the application to be submitted from institutions located in countries where the work is to be done.
	Deliverables are usually defined solely by projects completed or trainees taught during the project period.	Deliverables are defined as increased capacity with the primary goal being future sustainability.
Funds flow, disbursement,and management	Researchers from the funding country direct the project.	Researchers from the funding country provide technical assistance.
	The fiscal and administrative management is primarily performed by the donor entity.	Fiscal and administrative management is led from within the country in which the work is performed by a local institution (MIHER^2^).
	*Post hoc* distribution of funds.	Creation of and access to NIH^3^ PMS^4^ platform by the local administrator to allow withdrawal of money according to ongoing needs of the project.
Administrative infrastructure	Usually money is not available for training administrators for the project.	Training of Research Administrators to ensure good accountability is a priority.
	Fiscal and administrative support only for the specific project.	Fiscal and administrative support to assist researchers in designing and submitting financial reports.
	Limited interest in joining or creating synergies with other projects or sources of financial support.	Identification of other sources and partners to leverage research, training activities, and to strengthen the health care system.

Notes: ^1^MEPI: Medical Education Partnership Initiative; ^2^MIHER: Mozambique Institute of Health Education and Research; ^3^NIH: National Institutes of Health; ^4^PMS: Payment Management System.

**Table 2.** Contrasts between traditional model of collaboration and MEPI^1^ in what concerns monitoring and evaluation, faculty salaries and incentives, and collaboration and leveraging.

Monitoring and evaluation	Traditional model	MEPI model
	Focused on easily quantifiable units (generally samples or trainees).	Focused on capacity development.
	Moderate emphasis and fixed by the contract terms.	High emphasis and driven by changing opportunities and needs.
	Short-term projects.	Emphasis given to developing capacity in order to sustain long-term projects.
Faculty salaries and incentives	Funding agencies assume that the base salaries paid to researchers represent compensation for their full professional effort and are often not open to providing supplemental salary to reflect professional efforts on the project.	In the MEPI program supplemental salary supports (incentive payments) were made available to researchers and administrators from the South to purchase their time for research.
	Often, incentives paid to Southern researchers do not reflect the international value/cost of their work.	Incentives paid to Southern researchers according to the fractional time they spent and according to international standards.
Collaboration and leveraging	Local investigators are engaged in multiple separate projects, each of which is concerned only with its own project.	There is strong collaboration with multiple projects and researchers create synergies and learn from others’ experience.
	The projects and the personnel engaged have few connections to each other.	Networking with additional partners from North and South is strongly incentivized to ensure sustainability.

Notes: ^1^MEPI: Medical Education Partnership Initiative;

**Figure 2.**
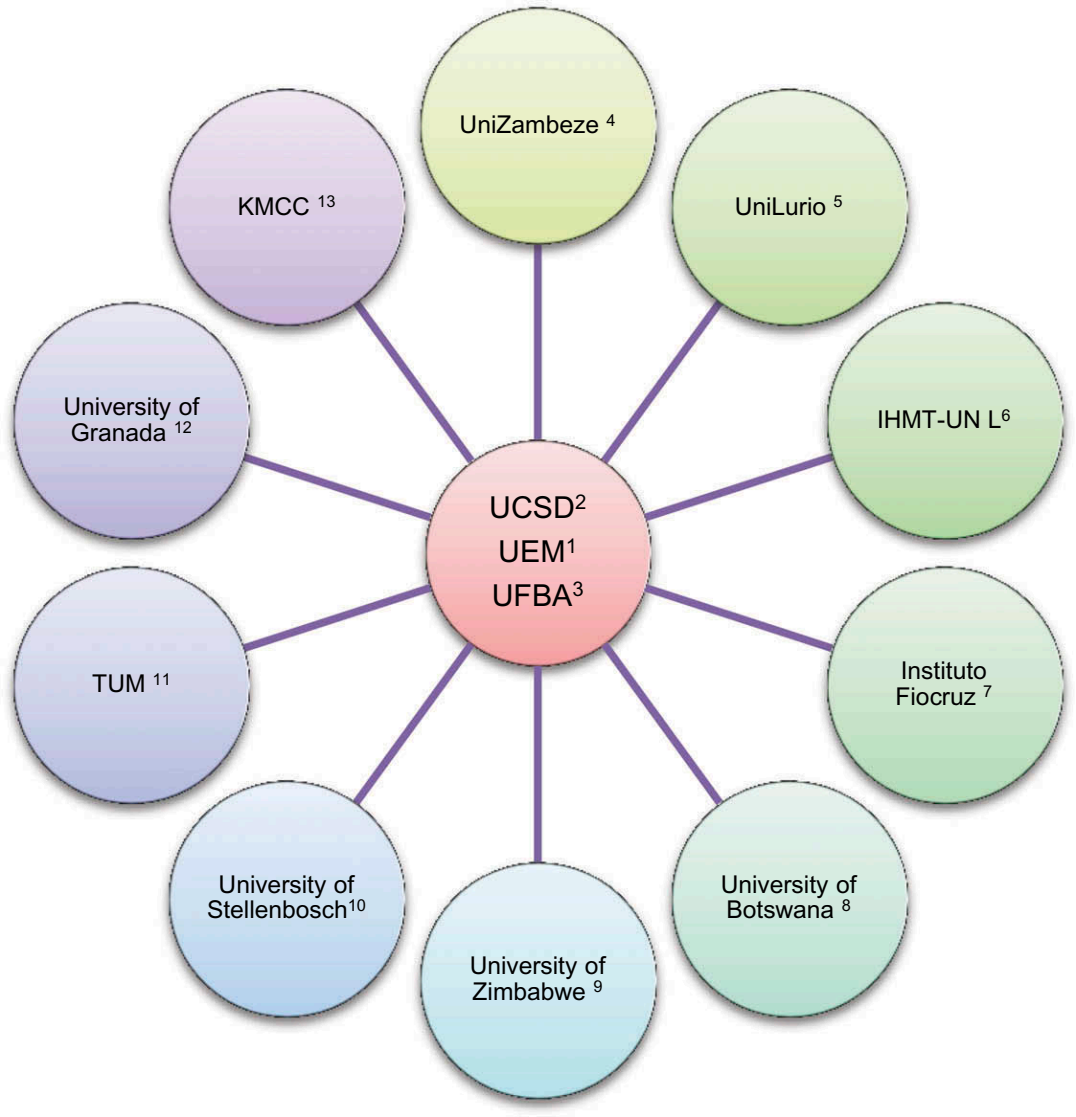
Collaborations rose within MEPI. Notes: ^1^Universidade Eduardo Mondlane, Mozambique; ^2^University of California San Diego, United States of America; ^3^Universidade Federal da Bahia, Brazil; ^4^Universidade Zambeze, Mozambique; ^5^Universidade Lurio, Mozambique; ^6^Instituto de Higiene e Medicina Tropical, Universidade Lisbon Nova de Lisboa, Portugal; ^7^Instituto Fio Cruz, Brazil; ^8^University of Stellenbosch, South Africa; ^9^University of Zimbabwe, Zimbabwe; ^10^University of Botswana, Botswana; ^11^Munich Technic University, Germany; ^12^University of Granada, Spain; ^13^Kilimanjaro Medical Christian Center, Tanzania.

### Definition of goals and deliverables

In traditional aid programs, a funding entity usually specifies specific deliverables, priorities, and/or training programs that it wishes to support in a request for applications (RFA) or funding announcement. Grant applications are often developed and designed primarily by entities within the granting country and local personnel are essentially ‘hired’ to execute the project if it is funded under the direction of the grant holder [5,7,9,22]. In many international capacity-building projects, the ‘deliverable’ is the number of research publications or health care workers who have attended a course or who receive a degree. Under this approach, when the project ends the increased output may end, as well.

In the MEPI model, rather than laying out specific tasks or aims, the RFA laid out programmatic goals and asked local investigators to develop specific aims that would best address these goals within the context of their own countries and institutions. The local investigators then identified international partners of their choosing to work in a collaborative and consultative way to outline specific research plans that were uniquely tailored to local needs. The bi-national teams then developed the applications and submitted them from their home institutions.

In the MEPI, deliverables are defined as increased capacity to train more health care workers and to improve the quality of those we train. Increases in production capacity and quality are enduring, will have an impact that will last for decades, and will result in a much greater cumulative impact than short-term traditional metrics of ‘number of health care workers trained’ during the project period itself.

### Funds flow, management principles, and methods of disbursement

Granting agencies generally have specific requirements related to accounting, taxation, procurement, and policies that are often in conflict with local policies and regulations under which programs must operate. These conflicts in spending restrictions and operating procedures may prevent programs from utilizing funds effectively [5,7,8]. In traditional models of collaboration, a ‘home country’ entity such as a university or a non-governmental organization (NGO) usually serves as the primary grant or contract recipient. These entities then subcontract for a local workforce through in-country institutions or as independent contractors. The MEPI model reversed the traditional model of funds flow. Medical schools within Africa served as the primary grantee. American collaborators were identified by the primary African grant recipients and issued subcontracts to provide technical assistance and other services as defined by the African leadership within the consortium. This fundamentally changed the nature of the relationship and ensured local ownership of the program. Local ownership of the MEPI program was an essential element of its success since its central goal was to introduce changes in medical education within participating in-country institutions. We found that vesting ownership locally was a major contributor to the willingness of critical stakeholders to embrace the proposed changes.

Another challenge faced by the MEPI relates to how research funds are disbursed. Many agencies sponsoring research or institutions through which funds flow to LMIC institutions establish inflexible ‘cost reimbursement’ payment systems. In these situations LMIC institutions are expected to bill sponsors or pass through entities retroactively for services performed and materials purchased. While ‘cash-rich’ institutions in Northern countries may have financial reserves that allow them to front the research costs and to be reimbursed later, institutions in LMICs rarely have sufficiently reliable cash flows to conduct business in this manner. This approach disrupts the research process and sometimes places research staff in the position of being without pay for months at a time [3,5,7].

In MEPI institutions, program funds were disbursed prospectively in anticipation of the conduct of the work and progress was assessed by a rigorous M&E program.

The system has evolved to use the US Government’s Payment Management System (PMS). Budgets were submitted annually at the time financial reports from the prior year were submitted to the program officer. Once annual budgets were approved money would be made available on the PMS through which programs could access funds according to ongoing needs within three business days. Whenever there was delay in implementing parts of the project and funds remained from the prior fiscal year, African institutions through their PIs could request a no-cost extension to finish the previously planned ongoing activities. This ensured that all funds were used smoothly and efficiently for projects outlined in the budget and avoided less than vital end-of-fiscal year purchases designed to capture unspent funds.

### Development of administrative infrastructure

The traditional approach of designating a primary grantee in the home country of the granting agency is often taken under the premise that institutions in LMICs do not have the capacity to manage large amounts of money. Funding agencies may be reluctant to allocate the resources required to strengthen administrative and management capabilities within the local institutions in which the research is conducted. In the traditional model, home country institutions may administer the funds in ways that do not fully take into account local priorities or take advantage of local opportunities. Residual funds at the end of budget periods may be sent back to the funding agency or expended by the primary grantee, although there are still many relevant unmet needs in the institutions at which the programs were initially directed.

Some of these difficulties are attributable to a lack of experience with grants management within local institutions. This inexperience can complicate the transfer of funds and the accounting of funds received and may provoke delays in project implementation. These deficiencies in grants management infrastructure may give rise to poor management and, on occasion, to misapplication of funds.

Administrative structures within primary grantee institutions in LMICs are often not structured to incorporate contemporary management principles of investigator-initiated externally funded research. This was the case for UEM and other Mozambican public universities in which operation and management principles are based on state public sector management principles. We thus created a research support center (the Mozambique Institute for Health Education and Research; MIHER, www.miher.org) to provide administrative and fiscal management of the MEPI grants. Seven UEM/MIHER staff completed research administration and grants management courses organized by the US NIH. Two additional program officers were trained locally to further augment management capacity. Staff development courses provided comprehensive training in grant and financial management and in research ethics administration, and positioned MIHER to provide administrative and fiscal support for faculty at UEM, UniZambeze, and UniLurio [11].

MIHER also provides support to researchers and junior faculty throughout the country in project design, grant preparation and submission, and administrative and fiscal management of externally funded research activities. Finally, MIHER identifies other sources and partners to leverage research, training activities, and to strengthen the health care system. MIHER has secured $5,716,175 for the next 5 years for research, training, and health care delivery activities to be carried out in the consortium universities and Ministry of Health. The funding structure facilitated efficient and transparent fiscal support and oversight and clearly demonstrated that the direction of the overall program originated within Mozambique. Since a primary goal of this project was to drive change within Mozambique’s indigenous institutions, it was essential to the success of the program that this effort be perceived in this way [11].

### M&E

In the traditional model of collaboration, M&E goals are often tied to the initial RFA and are established early in the project. Continued funding is assured only if the funding agency is able to verify that the metrics it established at the outset of the project have been met, whether or not the context or priorities have changed.

The MEPI model is much more dynamic and interactive in its goal setting and M&E process. The M&E metrics are based on the goals set for each project and are expected to be changed as project goals change and as new opportunities emerge. By deeply embedding the M&E process into the MEPI infrastructure, we both increased transparency within the project and built a vibrant culture of ownership and accountability that cannot be matched in externally imposed M&E models. This approach ensured the sustainability of the projects by clearly demonstrating local ownership.

### Faculty and staff salary considerations

Under the traditional model confusion may also arise around remuneration formulas for faculty and staff undertaking the work. Funding agencies routinely provide salary support to those conducting research in Northern institutions that reflects the fraction of their professional effort that is assigned to the project. The appropriate amount of support is relatively easy to calculate since faculty and staff in Northern institutions are generally appointed to full-time positions and their entire professional remuneration comes through their institution. Base salaries within Mozambican higher educational institutions (and many other Southern institutions) are set at levels that reflect only the direct teaching responsibilities of university faculty. Faculty members are expected to generate the remainder of their income from external activities. Consequently, faculty members may teach for several hours in the morning for a base teaching salary with the expectation that they will work for external agencies or in private medical practices for the rest of the day to generate the remainder of their income. When research grants are awarded, and these faculty members use these funds to reduce outside commitments, their institutions often refer to these funds as ‘incentives’ or ‘incentive payments’. These terms have a very different connotation in Northern institutions and may lead to confusion within granting agencies. Funding agencies often assume that the base (teaching) salary in Southern institutions is a full-time salary and do not understand that these ‘incentive payments’ are analogous to the fractional effort payments made to those working in Northern institutions [1,11]. The ability to access externally awarded research funds proportionate to their professional effort through their institutions as ‘incentives’ added to base pay, or through other pathways such as a dedicated research foundation, is critical if faculty are to redirect their external professional activities to funded research projects [2,11].

Addressing these features was essential to the success of the MEPI program in that it allowed local researchers to plan and direct the project and for them to be paid according to international standards. Those engaged in the project were totally committed to the project because of a sense of ownership and control and because they were paid in a way that allowed them to dedicate the professional effort required to successfully complete the project.

### Collaboration and leveraging

In the traditional model of collaboration, local investigators may simultaneously be engaged in multiple separate projects supported by several different entities – each of which is concerned only with its own project. The projects and the personnel engaged in these projects have few connections and as each one ends, little is left behind but a sample collection or a manuscript. In most cases, activities of the project involve only the funder; funded investigators and institutions have few connections with other programs. In the MEPI program, strong collaborations with our partners from the North were leveraged, in our case, to extend to collaborations with institutions in our own Southern region such as Stellenbosch University, the University of Zimbabwe, the University of Botswana, the Universidade Federal da Bahia, and the Instituto Fio Cruz in Brazil (see Figure 2).

Under the MEPI program, by emphasizing networking with peer institutions we have been able to borrow from the experiences of these institutions and to apply them in our own contexts. For example, we developed our eLearning strategies on the basis of experience of the Kilimanjaro Christian Medical Center (KCMC) in Tanzania and the University of Botswana. Many elements of our joint research and resident exchange program with UCSD have been adopted by the University of Zimbabwe. In addition to our individual and collective relationships, we have formed a MEPI Principal Investigator (PI) Council composed of the PI of each MEPI Program. This Council serves as a forum to chart the overall direction of the MEPI and has helped us to develop a vibrant network of people working together to improve medical education throughout the continent. This year in August the MEPI PI Council was transformed into the African Forum for Health Professional Education (AFRI Health) in order to incorporate other health professionals including those in nursing, pharmacy, and dentistry. This expansion was intended to support trans- and multidisciplinary training and research capacity development.

## The challenges

Our challenges are, in fact, many of the things the MEPI has been designed specifically to address. We continue to have too few trained health care professionals and trained faculty to implement MEPI goals. To overcome these challenges we continue to rely on the technical support from our UCSD partners as well as other collaborators from Portugal and Brazil, and other African universities. Only a limited number of Mozambican medical school faculty have had extensive exposure to the cultures of research and accountability. Salaries within our institutions are so low that external employment is required to meet basic living expenses. This further limits the effectiveness of our small workforce within our core institutions. We are challenged by the loss of some of our most talented people to local and foreign NGOs and the private sector that typically offer salaries two to four times those in the public sector [2].

We are also aware that Western economies have recently been struggling and feel it is critical that the investments made by our Western partners be ones that have the biggest and most long-lasting impacts. We continue to struggle with differences in requirements of granting agencies and those of our government related to accounting, taxation, and procurement. Finally, we are trying to make major changes in institutions in which we work but which we do not control. One of the critical features of the MEPI that has allowed us to change so many things in such a short period of time is that it is seen within Mozambique as a fundamentally Mozambican program. Had the MEPI not been perceived within Mozambique as being ‘indigenous’ we would have never been able to implement such substantial change so quickly.

## Discussion and conclusions

In this partnership between UCSD and UEM, African leadership and ownership was a clear priority from the beginning of the program. This was critical to our goal of ensuring the sustainability of the activities initiated under the program since they quickly became part of everyday activities. We have emphasized building local expertise and infrastructure to ensure that the goals and achievements of the MEPI will remain for decades. The collaboration was substantially strengthened beyond ties created by the MEPI itself during the course of the program through the development of a number of other externally funded projects that continue beyond the defined period of the MEPI and will further drive sustainability.

Although Mozambican institutions have been fortunate enough to receive substantial cooperative assistance from many different sources since our independence from Portugal in 1975, many of these collaborations have been challenged by some of the problems delineated previously related to grant and fiscal management. This particular investment has been unique and has allowed us to create a research support center (MIHER) and to train local research administrators to ensure proper management and accountability.

MEPI has nurtured a vibrant network of enthusiastic and committed medical schools. Faculty and staff engaged in the MEPI are working toward the sustainability of this initiative as well as toward extending the initiative to involve more schools and other health professionals. The resilience of the program is attributable to the visible progress each participating institution has witnessed since its inception and to the respect, transparency, and strong mutual interests upon which it is based. Ongoing communications and dialog among the main stakeholders (Ministries of Education and Health, medical professional organizations, funding agencies and partners, and our North American partner institutions) ensure the alignment of priorities and policies of each involved institution.

There are brighter signs today that Northern partners are willing to transform traditional North–South ‘collaborations’ into what are now often referred to as ‘true partnerships’. Interestingly, it is the Northern institutions that seem to lead the way. The Swiss Commission for Research Partnership with Developing Countries (2000) recently published guidelines for research in partnership with developing countries. The guiding principles were summed up by Gaillard [23] in his paper ‘North–south research partnership: is collaboration possible between unequal partners?’, which is now often quoted as the charter of North–South partners.

Although many research capacity development programs focus primarily on providing training to individuals [2], the MEPI program is strongly focused on strengthening institutional capacity in order to sustain longer-term progress by institutions that have been fundamentally transformed. Thus we emphasize building partnerships both with our international collaborators (in our case UCSD as well as several universities in Africa, Europe, and Brazil) and with local stakeholders. By doing this, we are able to leverage resources to achieve MEPI goals and develop funding streams that will last well after the term of the MEPI [11,19,24]. For example, at the inception of the program we defined increasing the number of postgraduate trainees as a major MEPI goal. When the Ministry of Health recognized the opportunity the MEPI was providing, it chose to more than double the size of Mozambique’s residency training programs [19]. The same happened when we created the Master’s degree programs at UniLurio, where the Vice Chancellor provided scholarships for faculty to enroll in the program and a university house for visiting professors.

The MEPI family also created communities of practice between the African countries and also US and European countries. Technical working groups were created to facilitate collaboration among African and US institutions. Those collaborations are made through webinars, annual symposia, joint academic writing workshops, and Skype meetings [7,11,18].

The MEPI program has had a profound impact both on the institutions it touched and on the approach to medical education in Mozambique. By investing in both human and institutional development, the MEPI will have a substantial impact on medical education and research and on the types of partnerships we will seek in the future that will live on for many years after the conclusion of the formal funding period. With these investments our universities are becoming research institutions that are equipped with the competencies needed to become increasingly vibrant and self-sustaining.

## Paper context

In this manuscript we critically examine different models of North–South collaboration, their objectives, and their evolution across decades, and compare these with the MEPI model of collaboration that has been implemented in 12 sub-Saharan countries in the last 5 years. Existing differences on the definition of goals and deliverables, fiscal and administrative management, faculty and staff remuneration, monitoring and evaluation, and collaboration and leveraging have been discussed and the authors conclude that the MEPI model is a paradigm shift in capacity building that should be followed for the future as it emphasizes building capacity based on mutual respect, joint interest, local priorities, and country ownership and leadership.
